# *NOD2* Polymorphisms and Their Impact on Haematopoietic Stem Cell Transplant Outcome

**DOI:** 10.1155/2012/180391

**Published:** 2012-10-18

**Authors:** Neema P. Mayor, Bronwen E. Shaw, J. Alejandro Madrigal, Steven G. E. Marsh

**Affiliations:** ^1^Anthony Nolan Research Institute, Royal Free Hospital, Pond Street, London NW3 2QG, UK; ^2^Section of Haemato-Oncology, Royal Marsden Hospital, Surrey SM2 5PT, UK; ^3^Department of Haematology, UCL Cancer Institute, Royal Free Campus, London WC1E 6BT, UK

## Abstract

Haematopoietic stem cell transplantation (HSCT) is a valuable tool in the treatment of many haematological disorders. Advances in understanding HLA matching have improved prognoses. However, many recipients of well-matched HSCT develop posttransplant complications, and survival is far from absolute. The pursuit of novel genetic factors that may impact on HSCT outcome has resulted in the publication of many articles on a multitude of genes. Three *NOD2* polymorphisms, identified as disease-associated variants in Crohn's disease, have recently been suggested as important candidate gene markers in the outcome of HSCT. It was originally postulated that as the clinical manifestation of inflammatory responses characteristic of several post-transplant complications was of notable similarity to those seen in Crohn's disease, it was possible that they shared a common cause. Since the publication of this first paper, numerous studies have attempted to replicate the results in different transplant settings. The data has varied considerably between studies, and as yet no consensus on the impact of *NOD2* SNPs on HSCT outcome has been achieved. Here, we will review the existing literature, summarise current theories as to why the data differs, and suggest possible mechanisms by which the SNPs affect HSCT outcome.

## 1. Introduction

Allogeneic haematopoietic stem cell transplantation (HSCT) is an important treatment option in the management of many diseases including malignant and non-malignant haematological disorders, immune deficiencies and inborn errors [1]. The increased knowledge of transplant biology and the effects of clinical factors and HLA matching have improved outcome. The primary choice of donor is usually an HLA-matched sibling, but the probability of a sibling being HLA identical is only 25%, a problem that is exacerbated due to small family sizes that are usually found today. Alternative allogeneic donor sources are thus often required and have now become an important and viable option. There are currently over 19.8 million volunteer unrelated donors (UDs) that have been recruited to registries around the world, with an additional 543,000 umbilical cord blood units also being available (as of September 2012) (http://www.bmdw.org/). The improvement in transplant techniques and practice has resulted in similar survival prospects for recipients of a well-matched UD as that using a sibling [2, 3]. However, the risk of posttransplant complications such as graft-versus-host disease (GvHD) and delayed immune reconstitution leading to infection is increased [4].

The vital role of HLA matching in transplant outcome is accepted, but there is still controversy as to which of the six major HLA genes are most important. The current perspective on what constitutes a well-matched donor is a 10/10 HLA allele match that is matched at an allele level for HLA-A, -B, -C, -DRB1, and -DQB1 [3, 5–7]. Comprehensive analyses of UD-HSCT pairs have shown that allelic mismatches are as detrimental to transplant outcome as antigenic mismatches, with a single allelic mismatch at HLA-A, -B, -C, or -DRB1 being associated with an increase in GvHD and a reduction in overall survival. This data has been confirmed in increasingly larger cohorts [8–11]. Mismatches at HLA-DQB1 appear to be better tolerated in the context of an 8/8 HLA-matched background (that is matched for HLA-A, -B, -C, and -DRB1) although there is some suggestion that they have a cumulative effect with any other HLA mismatch [6, 9, 10]. 

While the current donor selection criteria for matching donors and recipients usually refer to five of the classical HLA genes (HLA-A, -B, -C, -DRB1, and -DQB1), the impact of a sixth gene, HLA-DPB1, on the outcome of UD-HSCT is emerging. Current data suggests that nonpermissive HLA-DPB1 mismatches increase the risk of GvHD and transplant-related mortality [12–15].

Despite the benefit resulting from having a 10/10-matched donor, the survival of such a group of individuals is far from being absolute. Recipients receiving a graft from a well-matched sibling donor can be susceptible to getting GvHD. Conversely, some recipients of ≤9/10 HLA-matched grafts do survive and can achieve full remission of their disease [16]. While clinical factors such as the type of disease, disease stage, and recipient/donor characteristics are most certainly involved, theories have evolved that postulate a role for genes other than HLA in predicting transplant outcome. In recent years, much interest has been shown in the role of SNPs within innate immune response genes on the outcome of HSCT [17, 18]. One of the most prolifically studied genes to date has been the nucleotide-binding oligomerisation domain containing 2 (*NOD2*) gene (previously known as the caspase recruitment domain, family member 15 (*CARD15*) gene). The data from these studies is conflicting. Here, we will review the current data, on the impact of *NOD2* polymorphisms on the outcome of HSCT, potential causes of differences in the data and possible mechanisms by which the variants affect outcome.

## 2. *NOD2* Gene Structure and Function

The *NOD2* gene is located in humans on chromosome 16 (16q21) [19]. It is approximately 36 kb in length (35,938 bp) and encodes a protein of 1040 amino acids. *NOD2* encodes the NOD2 protein, a member of the NLR (NOD, leucine-rich repeat (LRR) containing) protein family [20–22]. Other members of this family include apoptosis protease-activating factor-1 (Apaf-1) and the MHC class II transactivator (CIITA) [23]. These proteins are classified by their common tripartite domain structure, namely, a central nucleotide binding domain (NBD, the NOD molecule), an amino terminal effector-binding domain (EBD), and a carboxy-terminal ligand-recognition domain (LRD). While all members of this family contain the central NBD region, the EBD and LRD differ between the different proteins. In NOD2, the central NBD domain is an NOD molecule which is surrounded by two CARD molecules (the EBDs) which enable recruitment of downstream signalling molecules and a series of 11 leucine rich repeats (LRR) which function as the LRD [24–26].

Early functional studies identified NOD2 expression in antigen-presenting cells, specifically intestinal epithelial cells [27], Paneth cells [28, 29], macrophages, and dendritic cells [21]. An increasing number of studies have demonstrated that NOD2 is expressed in a multitude of tissues including keratinocytes [30], T cells [31], NK cells, and CD34+ bone marrow stem cells [32, 33]. NOD2 is expressed within the cytosol and can be recruited to the cell membrane of intestinal epithelial cells [34, 35], a mechanism that appears to be important in the function of the molecule. Proinflammatory cytokines have been shown to regulate NOD2 expression [36].

The NOD2 protein functions as a regulator of infection by the recognition of pathogenic ligands and the induction inflammatory responses via a number of pathways. The most studied interaction is the response to the bacterial ligand muramyl dipeptide (MDP), a derivative of peptidoglycan, which is a component of both Gram-positive and -negative bacterial cell walls [37, 38]. Recognition of MDP by the LRD of NOD2 initiates a complex change in the structure of the molecule, enabling it to undergo self-oligomerisation via the NBD [25, 26, 39], and subsequently the recruitment of the effector molecule receptor-interacting, CARD-containing serine/threonine kinase (RICK) via homophilic interaction of their CARD domains. This recruitment of RICK by NOD2 causes the effector molecule to be activated, and initiates the downstream signalling events that lead to the induction of the nuclear factor (NF)-*κ*B and mitogen-activated protein kinase pathways [39–41]. In addition to this cytokine response initiated by bacterial infection, it has also been shown that upon exposure to MDP, NOD2 plays a key role in the initiation of the autophagy pathway [42, 43]. NOD2 has also been shown to respond *in vitro* to viral infection by the recognition of a single-stranded (ss) RNA ligand [44]. Here, ssRNA binds to the LRD of NOD2, but rather than recruiting the RICK as an effector molecule, NOD2 is translocated to the mitochondria where it is able to interact with the mitochondria antivirus signalling protein and initiates downstream signalling of the NF-*κ*B pathway. 

## 3. Genetic Polymorphism of the *NOD2* Gene

The *NOD2* gene is proving to be highly polymorphic with over 660 single nucleotide polymorphisms (SNPs) reported to date both in the literature [45–47] and in various online databases (http://www.genecards.org/, http://www.ensembl.org/ and http://fmf.igh.cnrs.fr/ISSAID/infevers/) [48–50]. The minor allele frequencies vary from less than 1% to over 30%, although significant differences between different ethnic and geographic populations have been demonstrated. 

**Figure 1:**
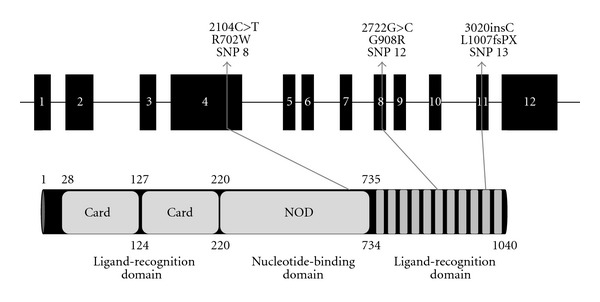
The structure of the *NOD2* gene and NOD2 protein. The numbering in the black boxes indicates the exon numbers. The numbering alongside the protein diagram indicates the amino acid positioning. SNPs 8, 12, and 13 are located within exons 4, 8 and 11 respectively, and encode either amino acid substitutions (SNPs 8 and 12) or a frame-shift causing early truncation of the protein (SNP 13).

Early studies to identify possible genetic factors that were affecting the incidence of Crohn's disease, a chronic inflammatory disorder of the gastrointestinal tract that can be complicated by anaemia, stenosis, and fistulae, mapped *NOD2* as a susceptibility locus [19]. Further studies identified three polymorphisms (designated nomenclature: SNP 8 (reference SNP (rs) rs2066844), SNP 12 (rs2066845) and SNP 13 (rs41450053)) as disease-associated polymorphisms (Figure 1) [45, 51]. It has been shown that individuals heterozygous for any of the three SNPs have a two- to fourfold increase in the risk of developing Crohn's disease, which increases to approximately twentyfold in individuals who are homozygotes or compound heterozygotes [52]. Other disease-associated studies have also tried to identify the impact of these three polymorphisms with varying results [53]. Subsequently, SNPs 8, 12, and 13 have become some of the most studied and well-characterised SNPs of the *NOD2* gene.

SNPs 8, 12, and 13 are located within *NOD2* exons 4, 8 and 11 respectively. SNPs 8 and 12 are nonsynonymous nucleotide substitutions that result in amino acid changes, SNP 8 (coding (c.) 2104C>T, protein (p.) R702W) and SNP 12 (c.2722G>C, p. G908R). SNP 13 differs in that it involves the insertion of a nucleotide that results in a frameshift within the coding sequence causing the introduction of an early termination codon and thus a truncated protein (c.3020CinsC, p. L1007fsPX). SNP 8 is located within the central NBD region of the molecule, while SNPs 12 and 13 are found within LRRs 7 and 10, respectively, of the NOD2 LRD [25, 46]. 

## 4. *NOD2* Gene Polymorphisms and Disease

Following the early studies in Crohn's disease, polymorphisms throughout the *NOD2* gene have been implicated in numerous diseases. SNPs 8, 12, and 13 have been correlated with increased risk of ankylosing spondylitis [54], psoriatic arthritis [55], and more recently with early-onset sarcoidosis [56]. Three additional polymorphisms, p. R334W, p. R334Q, and p. L469F, have been associated with Blau syndrome [57]. In addition to these inflammatory disorders, *NOD2* SNPs 8, 12, and 13 have also been correlated with an increased risk of malignant diseases such as colorectal [58], gastric [59], breast, and lung cancer [60] as well with the incidence of non-hodgkin's lymphoma [61], although in most of these studies, the detrimental effects of *NOD2* genotype were limited to the presence of SNP 13. More recently, *NOD2* SNPs have been shown to affect graft survival and mortality post renal transplantation [62] and coronary artery disease [63].

## 5. The Functional Consequences of *NOD2* SNPs 8, 12, and 13

SNPs 8, 12, and 13 are thought to reduce the ability of MDP to activate NOD2 and consequently the activation of NF-*κ*B, resulting in reduction in the production of cytokines and antimicrobial peptides [64–66]. These loss-of-function effects caused by the SNPs initially proved controversial, as an enhanced cytokine response is characteristic of Crohn's disease. The publication of data that showed mice with an *NOD2* variant similar to SNP 13 had increased sensitivity to MDP and elevated levels of NF-*κ*B activation when compared to WT mice suggested a gain-of-function effect of *NOD2* SNPs [67, 68]. While this evidence showed a plausible mechanism by which *NOD2* variants contributed to the onset of Crohn's disease, these findings have not been replicated in human studies, and further data has been published that confirm the loss-of-function mechanism [69–72]. Thus, the *NOD2* variants appear to reduce the ability of NOD2 to recognise MDP and consequently to stimulate NF-*κ*B responses. It has been suggested that the inflammatory response seen in Crohn's disease results from the inability of toll-like receptor-2 (TLR-2) to become tolerant to its ligand in the absence of appropriately functioning NOD2, resulting in upregulation of proinflammatory cytokines [73, 74]. In addition to these effects, SNPs 8, 12, and 13 have been associated with increased permeability of the gastrointestinal mucosa and consequently increased levels of bacterial peptides in systemic circulation [75]. 

The impact of *NOD2* variants other than the three aforementioned SNPs has not been investigated to the same extent. *NOD2* polymorphisms outside of the LRD do not appear to alter the ability of MDP to stimulate NOD2. In the case of the variants associated with Blau syndrome, all of which are located within the central NOD region of the protein, an increase in NF-*κ*B activity has been reported [25, 65]. This gain-of-function mechanism appears to be consistently demonstrated.

## 6. *NOD2* Polymorphism and the Outcome of HSCT

It was originally postulated that the *NOD2* variants that are purported to increase the risk and severity of Crohn's disease might also contribute to the risk of GvHD, particularly gastrointestinal GvHD, due to their notable similarity in clinical symptoms [76]. In the years following, many groups have published data on their attempts to test this hypothesis in a number of different transplant settings.  Table 1 summarises the differences in the cohort characteristics and the clinical observations reported by each group. 

In the first published study by Holler et al. [76] 169 HSCT pairs underwent *NOD2* genotyping for SNPs 8, 12 and 13. The cohort consisted of a mix of HLA-matched related donor, unrelated donor and a small number of one HLA antigen-mismatched related donor, transplants. Transplants were performed as a therapy for acute leukaemia, myeloproliferative disorder, lymphoma, or myeloma. Approximately 44% of the cohort underwent T-cell depletion, predominantly with antithymocyte globulin (ATG), while a small number of individuals were treated with alemtuzumab or CD34+ cell selection. The results of this study showed that 29.5% of HSCT pairs in this cohort had at least one of the *NOD2* variants. The authors correlated the presence of any of the three SNPs in the genotype of the pair (recipient, donor or both SNP positive) with increased severe aGvHD, (grades III-IV), severe gastrointestinal aGvHD and nonrelapse mortality [76]. When this was broken down further, severe aGvHD was increased in pairs with SNP-positive donors only, while an increase in severe and gastrointestinal aGvHD was described in pairs where both the recipient and donor were found to have any of the variants. This consequently increased the risk of nonrelapse mortality. 

In their subsequent analysis, the authors extended the cohort to 303 HLA-matched sibling HSCT pairs, transplanted at one of five European centres [77]. The underlying disease of the recipients was acute leukaemia, chronic leukaemia, bone marrow failure syndromes, or lymphatic malignancies. The authors did not report the use of T-cell depletion. *NOD2* genotyping of recipients and donors showed similar frequencies of SNPs 8, 12, and 13 to their earlier study and, importantly, between the different cohorts that were included in the study. The data showed that the effect of *NOD2* variants on clinically significant aGvHD (grades III-IV) and gastrointestinal GvHD persisted in this new cohort, while a trend for increased cGvHD was also noted. A dosage effect of the SNPs was seen in this study where individuals with increasing numbers of SNPs correspondingly had an increasing risk of aGvHD. The SNP dosage effect was also seen on the incidence of nonrelapse mortality. Survival was affected, but only when variants were present in the recipient genotype or in both the recipient and donor genotypes. The authors also described how the use of particular gastrointestinal decontamination agents could reduce the risk of aGvHD and nonrelapse mortality seen with *NOD2* SNPs. Specifically, the effects of *NOD2* variants were only seen in individuals who received either no decontamination or those whose protocol included the antibiotic Ciprofloxacin. 

In their third and most recent study, Holler and colleagues have extended their cohorts further to include 358 HLA matched related donor and 342 unrelated donor HSCT pairs [78]. Approximately 55% of the cohort underwent HSCT for acute leukaemia. The use of T-cell depletion varied between the two subgroups that made up the cohort, with 78% of cohort one (HSCT pairs from earlier studies) having some form of T-cell depletion included as compared to only 22% of cohort two (additional HSCT pairs). The impact of *NOD2* variant genotype was analysed separately in the related and unrelated donor cohorts. The presence of any *NOD2* variant in the genotype of the pair was correlated with significantly increased severe aGvHD (grades III-IV), non-relapse mortality and reduced overall survival in recipients of a related donor HSCT. In the UD-HSCT cohort, aGvHD was the only outcome affected by the presence of any of the three SNPs, while detrimental effects on nonrelapse mortality and survival were associated with the presence of SNP 13 within the donor's genotype. The association of specific gastrointestinal decontamination protocols (either none or Ciprofloxacin-based therapies) with increased effects of *NOD2* variants was confirmed in these cohorts.

Other groups have confirmed the effects of *NOD2* variant genotype on HSCT outcome described by Holler et al. A recent study by a group in The Netherlands described the effects of *NOD2* SNPs 8, 12, and 13 on the outcome of 85 HLA-identical sibling transplants [79]. The cohort included recipients with acute leukaemia, chronic myeloid leukaemia, myeloproliferative disorder, myelodysplastic syndrome, and lymphoma. The entire cohort had a partial T-cell depletion protocol included in their transplant protocols with the most common method being CD34+ cell selection. *NOD2* variant frequencies were similar to those reported in the earlier studies and in the general Dutch population. The authors confirm the detrimental effect of any *NOD2* variant on the risk of clinically significant aGvHD and nonrelapse mortality. As described in the earlier studies, the effect was most profound when both the recipient and donor were positive for any one of the SNPs.

Not all studies have been able to demonstrate an association of *NOD2* polymorphisms with GvHD. Elmaagacli and colleagues published data on the effect of the variants in a cohort of 403 related and unrelated donor transplants [80]. The recipients were transplanted for numerous diseases, predominantly acute leukaemia, chronic myeloid leukaemia, and myelodysplastic syndrome. Approximately 30% of the cohort had T-cell depletion included in the conditioning regimens either with alemtuzumab or with ATG. The frequency of *NOD2* variants in this cohort was similar to those described in other studies. Although an increased risk of aGvHD (grade III-IV) was seen when recipients and donors were both positive for one of the *NOD2* variants, a protective effect was associated with an SNP in the donor genotype. The protective effect was also seen on disease relapse in pairs where both the recipient and donor had at least one *NOD2* SNP. Unlike previous studies, no effects of *NOD2* genotype on nonrelapse mortality or survival were seen. The authors suggested that the possible reason for the lack of association here was due to their routine use of gastrointestinal decontamination with agents to target both Gram-positive and negative bacteria.

In a recent update by this group, the authors have investigated the affects of *NOD2* variants in a more homogeneous cohort [85]. *NOD2* genotyping was performed on a cohort of 142 AML recipients and their HLA-matched sibling donors. As in previous studies, the reported frequency of SNP-positive recipients and donors was similar to those found elsewhere. The cohort only included recipients who received myeloablative conditioning regimens and T-cell replete grafts. Unlike in their previous study, no protective effects of *NOD2* SNPs were associated with GvHD. A significant association was seen between SNP-positive recipients and an increased risk of any aGvHD (grade I–IV) and severe aGvHD (grades III-IV). Interestingly, after multivariate analysis, only a correlation with grade II–IV remained significant (relative risk (RR) 3.7652, *P* < 0.002). No impact on overall survival or nonrelapse mortality was reported.

Granell et al. also failed to correlate *NOD2* genotype with increased aGvHD [81]. Here, *NOD2* genotyping was performed on 85 HLA-matched sibling HSCT pairs. The underlying diseases of the recipients were acute leukaemia, myeloproliferative disorder, lymphoma, myeloma, myelodysplasia, aplasia, and chronic lymphocytic leukaemia. All recipients had T cell depletion included in their conditioning regimens, although the method was not reported. The authors report an association of recipient *NOD2* variant genotype with significantly reduced event-free survival. No other variable was significantly affected [81].

Our group has also reported the effects of *NOD2* genotype on HSCT outcome [82]. Here, the impact of *NOD2* genotype was investigated in a cohort of 196 recipients of an unrelated donor HSCT for an acute leukaemia. T-cell depletion was included in the conditioning regimens of 83% of recipients, with *in vivo* alemtuzumab being the preferred method. We reported a significant correlation between SNP-positive pairs (the recipient, the donor, or both had any *NOD2* SNP) and increased risks of disease relapse and death. In accordance with the data published by Granell et al. [81], we were also able to show a significant association with event-free survival. Interestingly, although the overall incidence of aGvHD was low in this British cohort due to the near universal use of T-cell depletion, a protective effect of *NOD2* SNPs on aGvHD was noted although it remained nonsignificant. Despite failing to achieve statistical significance, this data was in accordance to that reported by Elmaagacli and colleagues [80].

A study published in 2010 from a group in Dresden, Germany also reported a correlation between *NOD2* genotype and disease relapse [84]. This single-centre study included 304 HSCT pairs where the predominant diagnoses were AML/MDS (52%) and lymphoma (25.3%). Grafts were from either a ≥8/10 HLA matched unrelated donor (67.1%) or an HLA-matched related donor. Recipients receiving a graft from an UD had *in vivo *ATG included in their conditioning regimens. The authors performed extensive analyses to determine if an association between *NOD2* genotype and aGvHD could be identified. A trend towards reduced gastrointestinal aGvHD was reported in recipients positive for any *NOD2* variant, but this affect was limited to univariate analyses. There were no significant differences in GvHD in any of the other models tested. Recipients positive for any of the three SNPs did have a significantly increased risk of disease relapse, although this was only a trend after multivariate analysis (*P* = 0.056). 

A brief communication published last year highlighted the impact of *NOD2* SNPs in a large, multicentre, paediatric cohort [86]. A total of 567 HSCT pairs were tested. Donors were both HLA matched (78.7%) and mismatched (21.3%); the type of allogeneic donor was not stated. Transplants were performed for haematological malignancies, nonhaematological malignancies, and nonmalignant disease. The authors describe a significantly increased risk of nonrelapse mortality in recipients positive for SNP 13, an effect that persisted after multivariate analysis (RR 2.01, *P* = 0.049). This study also confirmed the effects of *NOD2* genotype on overall survival. A trend for lower survival was reported in pairs where the recipient had at least one of the three variants. Additionally, survival was also lower in recipients only positive for *NOD2* SNP 13. 

Two studies have specifically reported data on the impact of *NOD2* variants on bronchiolitis obliterans (BO) and bronchiolitis obliterans organising Pneumonia (BOOP), two serious late-onset, non-infectious pulmonary complications that can occur after HSCT. Hildebrandt et al. [88] analysed the incidence of BO/BOOP in a heterogeneous cohort of 427 HSCT pairs. Donors were either HLA-matched siblings or UDs. T cell depletion was included in the conditioning protocols of approximately 25% of the cohort although the method varied (ATG, alemtuzumab, or CD34+ selection). The incidence of BO was significantly higher when recipients, donors, or both were positive for *NOD2* SNPs, effects that persisted after multivariate analysis. It is important to point out, however, that the overall number of recipients who developed BO was very low in this cohort (11/427, 2.6%). In contrast to this data, Ditschkowski et al. did not find an association between *NOD2* genotype and the incidence of BO/BOOP in their cohort of 281 sibling donor HSCT pairs [87]. Transplants were for acute and chronic leukaemia, myelodysplastic syndrome, non-Hodgkin's lymphoma, idiopathic mnyelofibrosis, and multiple myeloma, and approximately 30% protocols included *in vivo* T cell depletion. As in the previously described study, the overall incidence of BO/BOOP was low (2.1% BO, 3.6% BOOP).

Despite the plethora of data available showing an effect of *NOD2* variants, several studies have suggested that there are no significant effects on HSCT outcome. Groups from Sweden [89], Germany [90], the United States [91] and The Netherlands [92] have performed extensive analyses in attempt to replicate the findings of the above-mentioned studies but have shown a lack of association with any of the outcomes measured. 

## 7. Discussion

There does not yet appear to be a consensus on the impact of *NOD2* variants on the outcome of HSCT. It would be reasonable to assume that the potential mechanisms of how the SNPs cause functional irregularities may be common but that the manifestation of the effects differs between groups. Here, we will discuss possible mechanisms by which *NOD2* genotype may affect HSCT outcome. 

NOD2 is known to function as a regulator of cytokine production and a mediator of proinflammatory responses upon recognition of the bacterial ligand muramyl dipeptide [40, 93]. Functional changes within the NOD2 protein are seen with SNPs 8, 12, and 13, all resulting in down regulation of cytokine production via the NF-*κ*B pathway [33, 94]. This dysregulation of cytokine production may provide the first mechanism by which *NOD2* variants can affect the outcome of HSCT. 

An early event posttransplant is the onset of the “cytokine storm” [95], an extreme increase in cytokine production as a response to both tissue damage in the recipient resulting from conditioning regimens and the activation of donor derived T cells to recipient alloantigens [96]. The result of the cytokine storm is the onset of both GvHD and graft-versus-leukaemia (GvL) responses [97, 98]. These tumour-specific cells are thought to be of T cell origin but data is emerging that suggest other cell types such as NK [99] and NKT cells [100] are also involved. One possible explanation of how *NOD2* genotype causes an effect after HSCT is that the inability of the NOD2 variant proteins to initiate cytokine production could, in theory, lead to a massive disruption of the cytokine storm, resulting in a lack of GvL or GvHD responses. 

While the effect of *NOD2* genotype-related dysregulation of cytokine production may not be the only contributing pathway to the cytokine storm, the role of NOD2 and other sensors of bacterial infection has long been proposed as major factors in GvHD responses. Studies that have shown that gastrointestinal mucosa damaged by aggressive treatments such as the conditioning regimens used in HSCT allow bacterial ligands, specifically the MDP homologue Lipopolysaccharide (LPS), to seep into systemic circulation. Once there, T cells specific for these ligands are capable of stimulating cytokine production and eliciting GvHD responses [101–103]. It has been suggested that *NOD2* SNPs can increase the permeability of the gastrointestinal mucosa and potentially increase the ability of bacterial ligands to enter systemic circulation [75]. It is possible that these events in combination with the inability of the variant NOD2 protein to respond efficiently to bacterial infection in recipients with *NOD2* variant genotype result in an increased level of circulating LPS, which are able to prime T cells and thus initiate strong GvHD responses. These effects are in concordance with the data published by numerous groups correlating *NOD2* variant genotype and increased aGvHD. 

NOD2 is also known to have a synergistic relationship with TLRs and is thought to provide some regulatory control over their ability to stimulate cytokine production [93, 104–106]. It is possible that the inability of variant NOD2 to regulate or be regulated by TLRs resulted in dysregulation of the cytokine produced, which in turn affected both GvHD and GvL responses. One of the most studied relationships is with TLR2 [107]. NOD2 is known to act as a regulator of IL-12 production via the simultaneous stimulation of NOD2 and TLR2 by their bacterial ligands with both positive and negative regulation occurring dependant on the dose of available ligand [104, 107]. Polymorphisms of *NOD2* are known to cause a reduction in IL-12 production [69]. Interestingly, in the context of HSCT, low IL-12 levels have been correlated with an increase in the incidence of disease relapse [108] without increasing the incidence of aGvHD [108, 109]. 

NOD2 is expressed both intracellularly and on the cell surface of epithelial cells. It has been suggested that this membrane recruitment of the protein is necessary to initiate a functional response [34, 35]. The repertoire of known cell types showing NOD2 expression is increasing, with both NK cells and CD34+ bone marrow stem cells recently being identified [32, 33]. It is thus feasible to assume that NOD2 is expressed on the cell surface of these other cell types. The presence of SNP 13 has been associated with the failure of the molecule to be expressed on the cell surface, although this has not been reported for the other polymorphisms [34, 35]. It is possible that the failure of leukaemic cells to express NOD2 extracellularly in recipients with *NOD2* variant genotypes results in their evasion of immunesurveillance activity. This escape mechanism would lead to the proliferation of leukaemic cells and thus disease relapse after transplant. This theory is consistent with the observations that *NOD2* polymorphisms cause disruption of GvL responses. 

Although no effect of *NOD2* SNPs 8 and 12 on the membrane recruitment of NOD2 has been reported to date, it is possible that they have an alternative mechanism by which they cause cells to evade immune responses. SNP 8 is located within exon 4 of the *NOD2* gene and is found between the NBD and the LRD of the protein [25, 110]. Self-oligomerisation of the protein occurs at the NBD, a process that is fundamental to the ability of the NOD2 protein to function [25, 111]. It is possible that SNP 8 causes a conformational change in the molecule rendering it either incapable of self-binding or causing it to function at a reduced capacity. Alternatively it may render the LRD either unable to or inefficient at binding its ligand. If this is the case, then it is feasible that even if NOD2 is recruited to the cell surface, it is unlikely to initiate a functional response that is adequate to initiate GvL effects. SNP 12 is located within the sixth LRR, which makes up the LRD [110]. The change in protein at this position may alter the ability of the *NOD2* molecule to recognise MDP, leading to the failure of NOD2 to initiate NF-*κ*B signalling and its related downstream events.

A logical explanation for the divergent results could be the heterogeneity in the characteristics and treatment of the recipients, not only between studies but also within each of the cohorts themselves. An obvious difference between the studies is donor source. The advances in transplant techniques and practice have resulted in similar survival prospects for recipients of a well-matched UD and related donor HSCT [2], suggesting that while donor source may contribute to the discrepancies in outcome associations reported, it is more likely that other characteristics of the cohort are correlated with outcome.

A second and strikingly different factor between the cohorts is the use of T-cell depletion within the conditioning regimens. T-cell depletion is used as a mechanism of reducing the risk of GvHD, although a consequence of this may be an increase in disease relapse [98, 112]. While most of the *NOD2* SNP association studies reported the use of T-cell depletion in their treatment protocols, several methods (alemtuzumab, ATG and/or CD34+ stem cell selection) were included, and thus it is important to consider the effectiveness of these different methods. For example, the anti-CD52 antibody alemtuzumab targets all human cells of lymphoid lineage, although NK cells appear to be relatively spared [113–115]. CD34+ stem cells are not targeted. Conversely, ATG functions by only targeting cell surface markers including those found specifically on T cells. B and NK cells are also targeted but only in excessive doses of ATG and are thus spared in most transplant protocols [116]. The effects of ATG are also long lasting which results in the specific depletion of T cells from the graft and any reconstituting cells. It is possible that the residual haematopoietic cells or indeed the lack of certain cell types present after different types of T-cell depletion could significantly affect the type and risk of post-transplant complication. 

In addition to the method of T-cell depletion used, notable differences in the number of recipients treated varied between the studies (approximately 30–100%). It is interesting to note that a high number of studies that reported a correlation between *NOD2* genotype and GvHD were either T-cell replete regimens or included ATG or partial CD34+ cell-selected grafts [76, 78, 79, 85]. Conversely, those studies that correlated *NOD2* variants with impaired Graft-versus-leukaemia (GvL) effects included consistently higher numbers of recipients treated with T-cell-depleted protocols (85–100%) and in some cases included alemtuzumab [81, 82, 84]. 

Gastrointestinal decontamination, a method of using drugs to control levels of bacteria within the gastrointestinal tracts, may also be used all around transplantation as a method of controlling GvHD [103, 117]. Holler and colleagues have suggested that the impact of *NOD2* SNPs may be more evident in recipients who received either no decontamination or those who were treated with Ciprofloxacin-based therapy [77, 78]. Elmaagacli et al. (2006) suggested that the lack of correlation between their data and that previously published could be attributed to their universal use of a decontamination protocol that includes a second antibiotic, Metronidazole, in combination with Ciprofloxacin [80]. In addition, the study by van der Velden et al. also highlighted the important role of bacteraemia in the outcome of HSCT in their study [79]. Unfortunately, most of the studies published to date have not included data on the use and/or type of gastrointestinal decontamination in their cohorts, and a few have analysed the effects of *NOD2* variants in cohorts stratified by protocol. It would be prudent for future studies to include this data in their analyses where possible in order for the exact relevance of this information to be obtained.

Several studies, including ours, have demonstrated the effects of *NOD2* genotype in recipients diagnosed with an acute leukaemia [82, 83, 85]. We have also reported on the lack of effect in recipients with chronic myeloid leukaemia in our cohort from the UK [118]. Other studies have not fully investigated the suggestion of a disease-specific effect. However, it is interesting that two of the four studies that did not correlate *NOD2* genotype with any posttransplant complication had a notably low number of recipients with acute leukaemia in their analyses [91, 92]. A possible explanation for this apparent disease specific effect is that *NOD2* SNPs alter the responsiveness of recipients with an acute leukaemia to their treatment. This may occur by modulation of the pathways of disease progression, rendering recipients resistant to treatment. While no direct evidence of the involvement of NOD2 variants in leukaemia progression exists, there is much data to show how it can affect the other diseases that are associated with the polymorphisms. In Crohn's disease, *NOD2* SNPs 8, 12, and 13 have been correlated with distinct disease phenotypes, in particular with the site of Crohn's disease within the gastrointestinal tract and with the age of onset [119–122]. *NOD2* genotype may also alter the recipient's response to drugs or conditioning therapies. Studies have shown that *NOD2* polymorphisms can affect the response to antibiotic treatment of perianal fistulas in Crohn's disease patients. The data showed that patients with an *NOD2* WT genotype had a 33% rate of complete response to treatment as compared to none of the patients with *NOD2* variant genotypes [77]. 

While the majority of studies have shown an effect of *NOD2* genotype on transplant outcome, data has been published that contradicts these findings [89–92]. As discussed, the lack of effect could be attributed to several characteristics of the cohort, namely the graft source, type of disease, use and method of T-cell depletion, and gastrointestinal decontamination. However, a notable difference between several of these studies and others published is the low incidence of *NOD2* SNPs reported. The overall SNP frequencies were between 10–15% lower than reported elsewhere. The difference in the frequency of *NOD2* SNPs between different ethnic and geographic populations has been widely discussed [123–127]. Thus, the low prevalence of SNPs in these cohorts may mask any affects that the genotype is having on transplant outcome.

A common feature of many of the studies is the correlation between recipient *NOD2* genotype and detrimental posttransplant outcomes. This may imply that cells which express NOD2 and remain in the recipient after their conditioning regimens, such as tissue macrophages, dendritic cells, and Paneth cells, may facilitate GvHD or GvL responses, and that these responses are limited in recipients with *NOD2* variant genotypes. The ability of recipient cells, specifically dendritic cells, to initiate GvHD effects has been reported [128]. Additionally, recently published data has demonstrated the importance of recipient *NOD2* genotype in murine models of GvHD [129]. Here, murine recipients of bone marrow and/or T cells from either wild-type (WT) or *NOD2* knock-out mice showed no significant differences in the ability of the repopulating cells to proliferate, to be activated, or on their expression of gut-homing molecules. The risk of developing GvHD was similar in the two groups. Conversely, *NOD2* knock-out recipient mice showed significantly higher levels of GvHD than their WT counterparts, and importantly, the organs targeted were the liver and the small and large bowels. Further tests showed that recipient *NOD2* genotype was also able to effect donor T-cell functional capabilities. While the translation of murine studies into human models does not always result in the same findings, these data in combination provide some evidence to substantiate the observation that recipient genotype appears to significantly correlate with HSCT outcome in humans.

The studies that have suggested the *NOD2* genotype results in impaired GvL responses do not fit this model. A possible explanation for this is that recipient cells that are more resistant to the effects of pretransplant conditioning regimens (in these studies, T-cell depletion in particular) are responsible for the lack of GvL effects. NK cells have been shown to be more resistant to the T-cell depletion agent alemtuzumab than other targeted subgroups [115]. The importance of NK cells in this model has been previously suggested [83], and their ability to function as tumour surveillance cells and mediators of antileukaemic responses is widely accepted [100, 130]. Importantly, it has been suggested that autologous NK cells can maintain remission in acute leukaemia patients, although this was described in the context of autologous transplants or chemotherapy induced remission [131]. NK cells have recently been shown to express NOD2 and also to be activated by the recognition of MDP by NOD2 in the presence of costimulatory molecules [32]. It is possible that this mechanism for NK cell activation is of critical importance in mediating early GvL responses after HSCT, but in recipients with *NOD2* variant genotypes, this NK cell activation is limited, resulting in a reduced ability to initiate GvL responses. Interestingly, in our study, where predominant T-cell depletion with alemtuzumab was used, an increase in disease relapse was seen in recipients with *NOD2* polymorphisms. 

Finally, it is important to consider what impact *NOD2* polymorphisms other than SNPs 8, 12, and 13 may have on HSCT outcome. It is possible that these SNPs are only markers for detrimental outcomes and that the true association is with one or more untested polymorphisms that may be in linkage disequilibrium with these known variants. As stated previously, *NOD2* is highly polymorphic with some minor allele frequencies reaching 40% in certain populations. It would be prudent for future studies to consider the effects of the previously unstudied variants in any future analyses. It is possible that reanalysis of the published data including novel variants may result in concordance between different groups and potentially elicit an effect of *NOD2* genotype in cohorts where no association has been demonstrated previously. 

Despite the many questions that remain even after eight years of investigation into the importance of *NOD2* genotype on HSCT outcome, it must be concluded that the gene and its variants currently indicate an important role in transplant biology. The published data also reaffirms the belief that personalised medicine based on a combination of recipient and donor characteristics, HLA matching, and non-HLA genetics could provide the key to superior outcomes after HSCT.

## Figures and Tables

**Table 1:** A comparison of the results published on *NOD2* genotype and haematopoietic stem cell transplant outcome.

Study	Donor source^a^	Recipient diagnosis^b^	T-cell depletion	Effect of *NOD2* SNPs
			Yes—*in vivo *	Increased severe aGvHD (gr. III-IV) in SNP-positive donors and pairs
Holler et al. 2004 [76]	Mixed	Mixed	43% WT pairs	Increased severe GI aGvHD with SNP-positive pairs;
			48% SNP pairs	Increased transplant-related mortality in SNP-positive pairs

				Reduced overall survival with SNP-positive recipients and when both recipient and donor are SNP positive
Holler et al. 2006 [77]	RD	Mixed	—	Increased transplant-related mortality with increasing numbers of SNPs
				Increased severe (gr. III-IV) and severe GI aGvHD with increasing numbers of SNPs

				Lower overall and severe aGvHD (gr. III-IV) with SNP-positive donors
Elmaagacli et al. 2006 [78]	Mixed	Mixed	Yes ~30%	Increased severe aGvHD (gr. III-IV) when both recipient and donor are SNP positive
				Reduced disease relapse when both recipient and donor are SNP-positive

Granell et al. 2006 [81]	RD	Mixed	Yes, 100%	Reduced disease-free survival in SNP positive recipients.

			Yes—*in vivo* Alemtuzumab	Reduced overall survival in SNP-positive recipients and pairs
Mayor et al. 2007 [82]	UD	Acute leukaemia	82% WT pairs	Increased disease relapse in SNP-positive recipients and pairs
			85% SNP pairs	Reduced disease-free survival in SNP-positive recipients and pairs

			Yes,	Reduced overall survival in SNP-positive pairs (sibling HSCT) and SNP 13 positive donors (UD)
Holler et al. 2008 [78, 83]	Mixed	Mixed	78% cohort 1,	Increased severe aGvHD (gr III-IV) with SNP-positive pairs (sibling and UD) and SNP 13 positive donors (UD)
			22% cohort 2	Increased transplant-related mortality with SNP-positive pairs (sibling HSCT) and SNP 13 positive donors (UD)

van der Velden et al. 2009 [79]	RD	Mixed	Yes, 100%	Increased severe aGvHD (gr. III-IV) in SNP-positive pairs
				Increased transplant-related mortality in SNP-positive pairs

Wermke et al. 2010 [84]	Mixed	Mixed	Yes, *in vivo* ATG for UD, 31.6%	Increased disease relapse in SNP-positive recipients (trend at MV)
				Trend for less GI aGvHD in SNP-positive recipients (UV only)

Elmaagacli et al. 2011 [85]	RD	AML	None	Increase in overall (gr. I–IV) and severe (gr. III-IV) aGvHD in SNP-positive recipients

Kreyenberg et al. 2011 [86]	Unknown allogeneic	Mixed	—	Reduced overall survival in SNP 13 positive recipients
				Increased transplant-related mortality in SNP 13 positive recipients

Ditschkowski et al. 2007 [87]	Mixed	Mixed	Yes, 30% *in vivo *	No significant effects on BO or BOOP

Hildebrandt et al. 2008 [88]	Mixed	Mixed	Yes, 37%	BO increased in SNP-positive recipients

Sairafi et al. 2008 [89]	Mixed	Mixed	Yes, 61%	No significant effects

Gruhn et al. 2009 [90]	Mixed	Mixed	None	No significant effects

Nguyen et al. 2010 [91]	UD	Mixed	None	No significant effects

van der Straaten et al. 2011 [92]	Mixed	Mixed	Yes, *in vivo* ATG for UD and HLA-mismatched RD	No significant effects

^a^Mixed as a donor source denotes both related (RD) and unrelated donors (UD) were used.

^b^Mixed as a recipient diagnosis indicates that the included recipients underwent HSCT for any one of a number of diseases.
